# Challenges in providing ethically competent health care to incarcerated older adults with mental illness: a qualitative study exploring mental health professionals’ perspectives in Canada

**DOI:** 10.1186/s12877-021-02687-9

**Published:** 2021-12-18

**Authors:** Kirubel Manyazewal Mussie, Félix Pageau, Helene Merkt, Tenzin Wangmo, Bernice Simone Elger

**Affiliations:** 1 Institute for Biomedical Ethics, University of Basel, Bernoullistrasse 28, 4056 Basel, Switzerland; 2 Center for Legal Medicine, University of Geneva, Geneva, Switzerland

**Keywords:** Older prisoner, Ethical issues, Qualitative, Mental health, Correctional health, Resource scarcity, Canada

## Abstract

**Background:**

The population of incarcerated older adults is the fastest growing demographic in prisons. Older persons in custody have poorer health as compared with those in the community. The unmet and complex health care needs of incarcerated older adults with mental illness raise justice, safety, dignity and fairness in care as ethical concerns. As there exists research gap to better understand these concerns, the current study aimed at exploring the perspectives of mental health professionals on challenges in delivering ethically competent care to mentally ill incarcerated older adults in Canada.

**Methods:**

Thirty-four semi-structured interviews were conducted between August 2017 and November 2018 with prison mental health professionals in Canada who were selected using purposive and convenience sampling techniques. The audio recorded interviews were transcribed verbatim and analysed inductively to generate themes.

**Results:**

The results were distilled into three main categories and seven subcategories that related to ethical issues in the provision of health care for mentally ill incarcerated older adults. The main categories included imprisoned older persons with special care needs, lack of resources, and the peer-support program.

**Conclusions:**

Results of this study showed that existing practices of care of mentally ill incarcerated older adults are characterised by challenges that increase their vulnerability to worse health conditions. It is imperative for local authorities, policy makers and representatives to prepare for and respond to the challenges that compromise ethically competent health care for, and healthy ageing of, mentally ill incarcerated older adults.

**Supplementary Information:**

The online version contains supplementary material available at 10.1186/s12877-021-02687-9.

## Background

Either by committing crimes at old age or staying longer in prisons, the population of incarcerated older adults is rapidly increasing and often reported as the fastest growing demographic [1, 2]. For instance, from 2000 to 2010, the overall population in US prisons increased by 17% while prisoners aged 55 and above increased by 181% [3]. This trend is similar for the incarceration rate in the UK [4] and is likely to be shared globally [5]. The definition of old age differs between the general population and prison settings. While the age used to define old age in the general population is 65 (at least in Europe and the US), old age in prison usually commences at the age of 50 due to the accelerated progression of chronic health conditions among imprisoned older persons [6–8]. As correctional facilities were never intended for older persons, provision of adequate health services to this group is a challenge worldwide. As compared with younger inmates and older people in the community, several studies indicate that older adults in correctional facilities have poorer somatic and mental health conditions [9, 10]. A review of literature on prison health care reports that “the main issues in prison health care are mental health, substance abuse and communicable diseases” [11], also shown in other studies on the topic [12–14], and those affected most by these health conditions in prisons are older adults [1]. Combined with the increasing number of older adults in prisons, the complex health care needs of incarcerated older adults burden correctional systems with resource needs, among other things [15].

In Canada, individuals sentenced for imprisonment for 2 years and more serve their sentence in federal correctional facilities administered by Correctional Service Canada (CSC), whereas those imprisoned for less than 2 years stay in provincial correctional facilities. CSC has five regional centres across Canada for offenders with serious mental health conditions [16]. There is evidence that most incarcerated individuals in Canada have mental health problems as defined by the Diagnostic and Statistical Manual of Mental Disorders [17, 18], and this is specifically true for imprisoned older adults among whom mental disorders are common [19]. In the face of such a challenge, the number of older adults (50 years and above) in the federal custody has increased by 50% over the last decade: between 2009 and 2019 [20]. For those between the ages of 50 and 59 only, number of admission increased from 382 in 2008–09 to 548 in 2017–18, representing a 43.5% increase [21]. In 2018, incarcerated older adults constituted 25.2% of the federally incarcerated population (3534 of the total 14,004 prison population number) [22]. The average age of individuals in federal custody has also been increasing and is now 40 years [20, 22]. This demography has brought, for example, more financial challenges on Canadian correctional facilities. CSC’s total expenditure in 2017–18 was approximately CAD 3.4 billion and the ageing prison population is a significant driver of rising costs [20, 23].

Although incarceration by itself has unique health risks such as substance abuse, depression and hygiene related diseases [12, 14], health can also improve in custody if, inter alia, the physical environment is made safe and comfortable for prisoners, human and physical resources are adequately available, and effective programs and strategies are designed [24, 25]. Custody might serve as an opportunity to offer health education and provide health care service to many people at once. There is evidence reporting prison interventions that resulted in, for example, reduced substance abuse and better mental health among incarcerated individuals [26]. In Canada where health care in correctional facilities is largely delivered by government authorities, there were better disease screening and treatment in prisons in the last few decades [18]. Despite these accomplishments, health care provision in Canadian correctional facilities continues to face challenges. CSC reports health care in Canadian correctional facilities as the most common area of offender complaint [27]. When it comes to mental health, screening tools in some correctional facilities do not sufficiently identify mental health disorders [28]. Mental health services for senior incarcerated adults are often described insufficient [19]. Moreover, “because of a lack of data, there is very little information known about the particularities of older adults’ mental health status in Canadian federal corrections.” [19]. The unmet and complex health care needs of incarcerated older persons raise justice, safety, dignity and fairness in care as ethical concerns. Moreover, such inadequate health care provision in correctional facilities conflicts with international standard rules for treatment of incarcerated persons, particularly, rule 25 of the United Nations Standard Minimum Rules for the Treatment of Prisoners (the Nelson Mandela Rules):

“Every prison shall have in place a health-care service tasked with evaluating, promoting, protecting and improving the physical and mental health of prisoners, paying particular attention to prisoners with special health-care needs or with health issues that hamper their rehabilitation.” [29]

As Canada’s Office of the Correctional Investigator states, in general, the vulnerability of older adults is not recognised by policies and legal frameworks, and this has implications for the care older adults receive in correctional facilities.

“There is no legal or policy recognition that older individuals represent a vulnerable population in prison or that they have unique characteristics, needs and rights which must be respected and met. As a result, their health, safety, dignity and human rights are not adequately protected.” [20]

Investigating the views and experiences of correctional health professionals working with mentally ill incarcerated older adults is considered a useful way to better understand and address the gaps in rehabilitating mentally ill incarcerated older persons [30, 31]. Despite the fact that mentally ill incarcerated older adults are the fastest-growing group among those in prisons and have the worst health condition [32, 33], mental health of incarcerated older persons “currently remains poorly researched and service provision for older prisoners with mental illness is poorly developed.” [34]. A recent systematic review recapitulates similar assertion and adds that literature is scarce about the mental health conditions of older people in prisons whereas literature is robust on the same topic but in communities [35]. Even though interest to study the health conditions of incarcerated older adults is increasing, studies on this area are not comprehensive [6]. In Canada, existing literature focuses on general prison population [36, 37], or comprises of reviews [18, 19]. However, to our knowledge, there are no empirical studies that delineating ethical issues in the care of mentally ill incarcerated older persons in Canadian correctional facilities. Thus, we addressed this knowledge gap by conducting a qualitative study involving mental health professionals working in selected correctional facilities in Canada with the aim of investigating the challenges in providing ethically competent care for incarcerated older Canadians with mental illness. By ethically competent care, we mean care given in a way that meets the moral and ethical standards in the context where the care is delivered. More specifically, as literature suggests, it is care that is characterised by safety, attentiveness and responsiveness to health needs, and by compassion towards the care receiver [38, 39].

## Methods

The overall aim of this study was to fill the gap in knowledge concerning older incarcerated adults in Canada by exploring health care providers’ perspectives on the challenges in delivering ethically competent health care for these adults with mental illnesses. TW and BE conceptualised the study. Both have considerable research experience and expertise in the areas of incarcerated older adults and bioethics [10, 40–42]. In conformity with the Human Research Act, this study received ethical approval from Northwest and Central Switzerland Ethics Committee (Ethikkommission Nordwest- und Zentralschweiz). Moreover, in Canada, this study received approval from Correctional Services Canada two times: one for data collection and another for publication. All methods were performed in accordance with the relevant guidelines and regulations.

### Participants

In total, 34 mental health professionals from Canada participated in this study (see Table 1 for demographic information). Employing purposive and convenience sampling techniques, we included participants who met a list of criteria and who are available for participation [43, 44]. The inclusion criteria include being a mental health professional and having substantial work experience in correctional context (minimum of 10 years work experience) in Canada. Individuals with no direct involvement in the care of mentally ill were excluded from the study. Some participants were recruited directly by one of the authors (HM) and others were recruited by Correctional Services Canada – a federal government agency responsible to incarcerate and rehabilitate convicted offenders in Canada.

**Table 1:** Demographic information of study participants (*N* = 34)

Professional experience	Number of participants	Participant IDs
Psychologist	7	MP1, MP2, MP3, MP18, MP26, MP27, MP31
Unit and case manager	5	MP9, MP12, MP13, MP14, MP30
Nurse	4	MP5, MP6, MP22, MP24
Chief of mental health services	4	MP19, MP23, MP21, MP32
Occupational therapist	3	MP7, MP20, MP28
Social worker	2	MP8, MP17
Research scientist	2	MP15, MP29
Legal coordinator	1	MP4
Director	1	MP10
Professor of criminology	1	MP11
Psychologist	1	MP16
Acting clinical team leader	1	MP25
Psychiatric nurse	1	MP33
Psychiatrist	1	MP34
**Gender**	22F, 12 M	

Participants were invited by email and/or phone. They received all information concerning the study and the informed consent form by email. During the individual interviews, the researchers explained again the study purpose and details in the informed consent. Then, written informed consents were obtained and participants were interviewed in person or via telephone. Study participants received no compensation. Compensating human research participants, even though it is an important practice, can act as a barrier to voluntary participation and good research efforts unless there is a sufficient reason to do it, which is actual cost of participation to the participant [45–47]. In our study, participants did not incur any costs because of their participation and thus, compensating them was not necessary.

### Data collection

We conducted semi-structured interviews, between 28 August 2017 and 14 November 2018, with mental health professionals recruited from health facilities such as regional psychiatric centres, CSC minimum security correctional institutions, and forensic mental health facilities. The interviewers were two research assistants (one of them was HM) who were working on their doctoral degree at the time of the interviews and were trained in qualitative interview techniques. In addition to background information and work experience of participants, the semi-structured interview guide focused on the mental health professionals’ perspectives on incarcerated persons’ health care needs, availability of resources to facilitate quality care to imprisoned older adults with mental illness, and existing medical practices and programs posing ethical concerns in the care of mentally ill incarcerated older adults (see Additional file 1). The interview guide was updated as the interviews progressed, adding probing questions wherever necessary.

The data collectors met the interviewees for the first time on the day of the interviews and thus, there were no relationships between interviewers and participants prior to data collection. The interviews were conducted one-on-one either in-person or via telephone. Each interview took approximately 1 h on average – the longest taking 92 min and the shortest 21 min. We audio recorded all interviews upon verbal consent from participants. In addition, the interviewers were taking some notes during the interviews to capture non-verbal aspects and facilitate probing. The number of study participants, and of interviews, was determined by the level of data saturation [48].

### Data management and analysis

The audio-recordings were transcribed verbatim and checked for quality and accuracy by two research assistants supporting the project. All identifying information were anonymised to assure participants’ confidentiality. We used the MAXQDA software to facilitate and manage the data analysis process. To analyse the interviews, we used Braun and Clarke’s thematic analysis framework consisting of six phases of analysis: familiarising with the data, generating initial codes, searching for themes, reviewing themes, defining and naming themes, and producing the report [49]. We used an inductive approach to code our data. Five members of the research team met to code 4 transcripts together in order to ensure a consistent procedure of giving code names and defining the meaning of complex code names. Subsequently, another team member coded the remaining transcripts and discussed them with TW to solve disagreements and prepared the final coding tree. The use of codes and code naming was once again checked by TW for accuracy. The analysis resulted in the development of three issues within the aim of the study: (1) complex health care needs of incarcerated older adults; (2) lack of resources to address their needs; and (3) ethical challenges with involving peer offenders. The results were discussed and agreed upon with the co-authors. We have included some quotes from the transcripts in order to illustrate these themes.

## Results

The thematic analysis of the transcripts led to three key themes that present the challenges in providing ethically competent health care for incarcerated older adults with mental health illness in Canada. These themes and their subthemes are presented in Table 2 and are explained below.

**Table 2:** Themes and subthemes

Themes	Subthemes
Imprisoned older persons with special care needs	Prisons - not suitable for older adults
	Imprisoned older adults’ emotional care needs
Lack of resources to ensure necessary care	Inadequate housing conditions
	Limited manpower and expertise
The peer-support program in prisons	The role of peers who help older adults in need
	The mutually benefitting outcome of the peer-support program
	The ethical concerns of the peer-support program

### Imprisoned older persons with special care needs

#### Prisons - not a place for older adults

In light of vulnerability and complexity of treatment, participating health professionals often noted that incarcerated older adults have more complex health conditions. Due to this, participants lamented on the incarceration of older adults in general.

*“I find it very sad to see a person of, for example, 80 years old incarcerated. The image is difficult. It evokes our empathy even more. It is very sad to walk around in this establishment because we see many elderly people in wheelchairs, with walkers, with canes, … it is a troubling image.” (MP19).*

In the same vein, other interviewees added that the forensic system in general is not set up in a way that meets the special care needs of older adults. One participant elaborated this challenge by making special reference to dementia.

*“We have clients who are ageing. Our staff is not necessarily trained to provide care to individuals with dementia. Our systems are not set up that way; our units are not set up that way. The forensic system is not really set up that way. Someone with dementia is going to get worse and worse and not better and better. We know that. Their dementia is going to worsen, right? And our system is not necessarily designed to address that. We treat them like anybody else.” (MP13).*

While some participants often asserted that incarcerated older adults should not be imprisoned as they mostly suffer from complicated health conditions, they also added that prison settings need to improve to provide adequate health care services to older inmates. It was argued that older adults in a prison setting require extensive medical care. Our participants mentioned that regardless of the type of their health conditions, imprisoned older adults are often times lower functioning who require assistance with their activities of daily living.

*“Some of them [incarcerated older adults] have mental health illness, some of them do not. But they generally are lower functioning groups that need help with their usual activities of daily living. So, they might need help bathing, showering, cleaning their room.” (MP21).*

Further explaining why prison is not a place for older adults, our participants particularly underlined their mental and somatic health conditions. For instance, some health professionals mentioned cognitive impairment and further explained how meeting the health needs of imprisoned older adults with cognitive impairments can be complicated and challenging.

*“Well some people have cognitive impairments. You know these problems were not there ten years ago and might have started five, two or three years ago. And now the first thing is realising that there are some impairments, realising that you will have to repeat the instructions more often, realising that you will have to make sure that the person understands that it is not about judgement but a new reality for these people. … their capacity has diminished somehow you know.” (MP10).*

All participants highlighted that imprisoned older adults have physical health conditions that necessitate extensive medical services. First, it was argued that comorbidity characterises the health care challenges of incarcerated older adults: *“As people get older their bodies wear out more and they are more susceptible at older age to cancer and other kinds of physical health issues.” (MP30); “Our older folks are going through health issues like diabetes or heart problems.” (MP31).* Regardless, the physical health care needs of incarcerated older adults were emphasised. Second, participants also added that some incarcerated older people have physical disabilities that affect their mobility and make them in need of extensive care: *“Well we have guys that are 24 hours in need. They are usually elderly; they have mobility issues.” (MP24).* As another participant added, incarcerated older adults with physical disabilities require personal assistance with their movement within their physical environment even though they have assistive devices such as walkers and wheelchairs.

*“We have older offenders who cannot walk upstairs, we have offenders who cannot get across the institution without having somebody assist them. So, they need somebody to walk beside them as they use their walker, or they need somebody to actually push their wheelchair.” (MP25).*

In further describing the complex health care needs of incarcerated older adults with mental illness, interviewees noted that some suffer from both mental and physical health challenges. In such cases, it was argued that these patients need 24-h nursing care: *“We have a population of offenders here who have mental illness but are also aging, so they have a variety of physical health disabilities that require 24-hour nursing care” (MP20)*.

Our participants also added that the mental and somatic conditions of the incarcerated older adults are highly intertwined with each other. A lack of comprehensive care is often reported. As one health professional highlighted, it is important to see the older prisoner as a whole and to give equal attention to both physical and mental health challenges.

P: *“I would hope we have a specialised care because some of the comorbidities that they [incarcerated older adults] have, whether it be diabetes, respiratory issues, seizures, cancer, etc. I mean without mental health there is no health, right? There is a mind that is not thinking clearly or appropriately, but there is a body still attached to that mind and it's all together and it can impact how they are feeling mentally, how they express themselves and how they engage. It's not a disconnect at the shoulders, right? And I think sometimes that's forgotten. That there is a human being here who has many functions going on, and their thinking can affect their sleep, can affect their appetite, can affect everything.” (MP12).*

I: *“Sometimes it does get forgotten?”*

P: *“Yes. I think sometimes people would concentrate on the mental health and, you have to remind them that there is this medical, surgical aspect of how a human being functions: their kidney function, their bowels, their appetite, the physical activity. And we encourage all of those activates.” (MP12).*

#### Imprisoned older adults’ emotional care needs

Our participants described most of incarcerated older adults as lonely who are disconnected from their connections with families and friends: *“They do not have family come in and visit them. It must be really lonely to have your family not visit you or any friends coming to visit you. That has got to be lonely.” (MP31).* Thus, “*they* [incarcerated older adults] *need that emotional support to feel safe and respected” (MP12).* The emotional support these individuals need was elaborated further by some of the health professionals. Our participants emphasised that the ability to understand emotions and manage them are important skills older inmates need to acquire: *“They need to learn skills, they need to learn how they manage their emotions, they need to learn how to recognize their emotions and deal with them.” (MP28).*

Within the framework of emotional support need of incarcerated older adults, participants also discussed whether they (or the correctional facilities in general) have the responsibility to facilitate conditions that improve the emotional wellbeing of older inmates. They explained this by highlighting that they have the responsibility to make emotionally challenged incarcerated older adults feel respected and worthy.

*“My work is about recreating emotion or social connection with people. It is to allow them to engage in relationships that are healthy, accepting and non-judgemental. And providing encouragement and uh hope for what their life could be like. A lot of them [incarcerated older adults] are disconnected from their families or their friends and they are only hanging out with each other. So, they become very isolated to our hospital. So, a big part of my role as a clinician is to help them have normative relationships with people that they wouldn't otherwise have.” (MP15).*

*“There is emotional component of what I work for them [incarcerated older adults]. As a nurse, I am helping them feel valid and worthy.” (MP12).*

Some participants expressed the fear that lonely and emotionally challenged older inmates could inflict harm on both themselves and others around them. If loneliness is allowed to sustain and these people do not get support to address their intimacy needs, the consequences could range from being violent towards others to committing suicide.

*“We know loneliness is very strongly related to aggression towards others, so if they are not meeting their intimacy needs effectively they are much more likely to commit violence towards others.” (MP1).*

*“I was looking at some data. The highest rate of suicide is among 80 plus year-olds. Because of the lack of hope. Because they don't think that they are worth it or that it's worth living, looking at depression and especially when their family members are not available, or they've lost them due to maybe a crime that they committed. It's very complicated.” (MP12).*

### Lack of resources to ensure necessary care

Lack of resource was a dominant theme in our participants’ viewpoints. Health professionals working in prisons described their work environment as lacking the housing, human and knowledge resources needed to meet the health care needs of incarcerated older adults with mental illness.

#### Inadequate housing conditions

Interviewees often reported that the physical environment is inadequate to meet the health needs of incarcerated older adults. One manifestation of such inadequacy is the size of the cells and the challenge that poses on the work of health professionals:

*“Some of our cells are so small that there is only one spot where the bed can actually fit and then if the prisoner needs assistance with transferring, it is difficult for the nurses. Some of them have hurt their backs trying to get older folks out because of the placement of the bed in the cell.” (MP20).*

Another participant added: *“The cells are very small for them* [incarcerated older adults]*. And the fact of having a lot of stairs or steps to go up or down becomes problematic.” (MP22).* As this participant continued to explain the situation further, we came to understand that this problem is far from being solved because of resource constraints: *“We can’t demolish and rebuild, that is impossible. The budget doesn’t allow it, but I don’t know when it is going to be possible. In fact, we would have to change the clientele.” (MP22).* This concern was echoed by another participant who, referring to the size of the cells, emphasised the ethical implication this has on the way prisoners are treated: *“It is inhuman because the cells are tiny and then two people in the same cell who live there for 15 years, it is somehow inhuman.” (MP19).*

Such an inadequate facility appears more challenging when it comes to working with incarcerated older adults with complicated health conditions. Interviewees voiced concerns about the inconveniences this brings to the older inmates who have chronic health conditions:

*“All three of the cases that I mentioned: the guy who had the persistent urinary tract infection, the one with traumatic brain injury, and the one who was in the wheelchair. All three of those guys were lifers. This kind of kicks off some internal thing for me when they are in a life sentence and we cannot seem to meet their needs. This makes me ask myself "what are we really doing here?". Because we have lifers who are aging, all of them are over 60, who likely will never go back to the community, just because of where they are at in their sentence or where they are at in their life. So, what are we going to do with them, right? We do not have a place to house them that seems to meet their needs.” (MP17).*

#### Limited manpower and expertise

Shortage of manpower was often mentioned by our participants as one of the gaps within this theme. As one participant said: *“Generally I would say more staffing is probably what we would need.” (MP7).* Another health professional added, referring to the peer program which involves engaging better functioning inmates to provide care for the less functioning ones,

“*I would say the reason why we have the peer program is because there's not really any help for care. Twice a day and half a week is really not enough to give them [incarcerated older adults] basic care, a shower, nail clipping, etc.” (MP22).*

Interviewees often explained the human resource scarcity by making references to specific professions. For example, participants voiced their concern about the lack of mental health professionals and its limitation on the mental health services to prisoners. The mental health needs of inmates are disproportionate to the available manpower, which resulted in the absence of mental health services such as counselling. Some interviewees referred to some mental health issues such as trauma and described the lack of resources to address them. One occupational therapist who works with older offenders with mental health problems was concerned about the shortage of mental health professionals such as psychologists, social workers and occupational therapists, and about the implication that has on the quality of mental health services provided to incarcerated older people:

*“I would say that the primary issue is staffing. So just to give you an example here, we have got 55 guys and we have got one psychologist. That psychologist obviously does not have time to do counselling. That psychologist is doing risk assessments, occasionally cognitive assessments and diagnostic clarification assessments. There is no time for counselling. I think that this is a common issue here that right now we are really short staffed, we are not in a good place. One psychologist, one social worker and one occupational therapist, this is our clinical team [laughs]. So, it is challenging, right? There are not enough mental health workers.” (MP28).*

Similarly, from the participants’ point of view, there are not enough human resources to care for incarcerated older people with cognitive problems such as dementia. Some of our participants reported that they did not encounter a cognitive or neuropsychological issue that they were unable to manage. However, they imagined how difficult it would be if that were the case. If there were incarcerated older adults with serious neuropsychological problems, as one interviewee emphasised referring to a specific correctional facility, prison settings would have difficulty providing the needed services. When asked to reflect on the difference between the health services provided to young prisoners and those provided to the old prisoners, this participant replied the following:

*“They* [incarcerated older adults] *have access to the same services. In recent years, I cannot say that I have seen cases there of elderly inmates who needed very, very, very [sic] special care that we wouldn't have been able to offer them. But of course, we lack resources in neuropsychology, for example, so I could very well imagine that if we had several elderly inmates who required, for example, an assessment of cognitive functions because we suspected a dementia or degenerative process, we would be a little confused, we would be a little limited because we don't have enough resources in neuropsychology.” (MP23).*

Whenever human resources are available, a gap still exists as the available health professionals do not have specific knowledge for health problems affecting incarcerated older adults. This is especially the case with addressing the needs of incarcerated older adults with cognitive or neurological problems, whom some of our participants refer to as “psycho-geriatric prisoners”:

*“We have correctional officers who are coming but not trained to work with psycho-geriatric prisoners. So, they are disoriented by it; it is confusing; it is difficult. I am hoping that in the new policy development, some of the things that are addressed are not always the management or the offenders, but the training and support for staff and just creating different systems to support the population* [psycho-geriatric prisoners]*.” (MP25).*

*“Even our health care staffs do not really have a good understanding of things like dementia and how it would impact incarcerated older adults in their functioning. Knowledge about what we would be looking for as signs of dementia and how we would recognise it and all such things.” (MP28).*

Considering the need to address the existing knowledge gap, another health professional added that staff training on mental health is a necessity in order to handle, for example, dementia, trauma and personality disorders:

*“It is a lot of education I think that needs to happen overall. So, staff training is needed, not just on dementia but on mental health in general. And staff training on the rehabilitation approach and the recovery model. What does that mean, what is trauma informed to me, why do we need to have a trauma informed lens, and what impact has trauma on prisoners’ behaviour. We also need education of personality disorders.” (MP28).*

### The peer-support program in prisons

#### The role of peers who help older adults in need

The peer-support program is another theme that emerged from our analysis. Eleven of our participants talked about it. The peer support program is designed to create better living conditions for persons with functioning impairments, particularly for older inmates with mental health problems and others with somatic health complications. These inmates can obtain help from better-functioning prisoners, who receive remuneration for the work they do: *“They [older inmates] are all assigned a peer helper, so another inmate has a paid position as their paid helper.” (MP22).*

These peer helpers could be younger offenders who do not have mental health problems or other serious health conditions and who volunteer to be caregivers. They take a variety of responsibilities ranging from assisting older inmates when they move from one place to another within the correctional facilities, and providing companionship when the older inmates need someone to talk to. A few of our participants also commented on the workload the peer caregivers have. The peer helpers are available anytime to assist whenever an incident or emergency happens.

*“The peer helpers make sure that the inmates get to meals. If they are in a wheelchair, they will push them around. If they need some minor help with their physical care, they can help with those types of things.” (MP27).*

*“Well in terms of mental health there is a lot of need. We have a peer support program where peers check on anybody who wants to be talking with somebody that is a peer. So at this place we have peers who can be called at any point in the day or night to talk to other offenders if they feel isolated, alone, depressed, anxious, etc.” (MP30).*

The peer helpers get some training helpful for their work as care givers: *“They do get like an in-house formal training. It is called “peer-assist training“ or something.” (MP27).* Two participants further elaborated on the training content, as well as information about the screening and interviews of peer caregivers as part of the recruitment process.

*“The peer caregivers are doing special dementia training to understand better the peers they are helping.” (MP18).*

*“We have something called the "the peer-assisted living program" here. So we train inmates whom we have screened and interviewed. We have created our own training materials which is, for example, about the endocrine system, transferring clients, etc. The inmates we choose for this are appropriate because they have finished programming inside the institution and they have shown a period of stable behaviour.” (MP25).*

### The mutually benefitting outcome of the peer-support program

Explaining the tasks peer caregivers perform, some of our participants specifically highlighted the benefits of the peer-support program. One important advantage most of these participants underlined was a long-lasting and positive relationships which incarcerated older adults benefitted from. Since the peer caregivers live in the institutions, their connection with the older adults lasts long and creates a sense of family.

*“I think this place have given them that sense of purpose and family. And so the clients do not have anybody else in the world, outside of these walls, and many of the caregivers do not either. So they care each other deeply.” (MP25).*

*“This peer program has some positive effect in the sense that it brings is a real familiarity and a relationship that forms between the one assigned care giver and the one older inmate that they take care of. That is a daily and long-term thing so I think that might help as opposed to, for example, in the community seniors care facilities or nursing homes where there might be shifts that change and different care givers come in on different days. So one big advantage of this peer-support program would be that you have a stable relation with your care giver.” (MP34).*

Furthermore, the peer caregivers were described as taking a teaching and mentoring role. One participant told the interviewer that the peer helpers teach the older inmates about, among other things, taking care of their chronic health issues and doing physical exercises.

*“The peer-support program is very, very helpful. The peer helpers not only just do the personal care but are also mentoring and teaching the older folks the right skills depending on who they are looking after. They teach them things like how to look after or better manage their diabetes. Moreover, they assist them to have a better meal plan and encourage them to get out and get some exercise.” (MP33).*

Finally, a few participants also emphasised the benefits of the peer-support program to the peer caregivers themselves. It was argued that this program contributes towards a positive prison environment as it helps the peer careers improve their behaviour and develop higher self-esteem. Two participants noted the following:

*“I can say that there are many many times I am blown away. Because we have caregivers who have committed some very atrocious crimes and historically would be individuals you would look at and not think to show empathy. And they are very caring and very invested. It has been amazing to watch these men show such empathy, care and investment.” (MP25)*

*“I think it is still important to have that intergenerational mix because the young learn from older people and older people learn from younger people. We learn from each other. The peer caregivers are actually benefiting tremendously from a rehabilitation point of view. They benefit tremendously because they feel like they are making a difference and they are doing something for the community by helping other people. So, this helps them develop their self-esteem and improve their mental health. So this is a win-win situation.” (MP28).*

### The ethical concerns of the peer-support program

The peer-support program, despite its benefits, poses questions and challenges. Of the 11 participants who talked about this program, four were concerned about whether it is ethically justifiable to make one offender look after another. These participants voiced their concern about whether it is right to rely on information provided by one inmate about the health conditions of another.

*“As a psychiatrist I wonder about the ethical issues around the peer support program. Because you have essentially another offender bringing patients into the appointment with you. And you as a psychiatrist or a mental health care provider are relying on another offender to provide you some collateral information about their day-to-day functioning or what might be the issue. In some ways we do want that information and somebody who spends a lot of time with these individuals presumably has a lot of observation and first-hand information. But what are the ethics of relying on another offender as opposed to a staff member? This is where the dilemma comes in for me.” (MP34)*

Explaining their scepticism towards the rightfulness of involving offenders as caregivers to older inmates, participants pointed to the knowledge competence of the peer helpers. The peer caregivers’ knowledge was judged limited as compared with the responsibilities they have. Our participants also added that the act of involving unprofessional (inadequately trained) labour to do professional work is the results of the health authorities’ effort to save money. Had it not been for this reason, it was argued, it would have been possible to hire more mental health professionals to do the work.

*“The peer caregivers are no professionals basically. It seems like a cope out on part of the system to save money. Instead of employing 10 or 20 care aid workers who might cost a substantial amount of money, they have these peer caregivers who they pay literally less than a dollar or a dollar-something per day to do the job. … They look after the older inmates without much training. For instance, in a community-based nursing homes there would be at least a trained health care aid who would be looking after these individuals. But here we have another peer offender who for whatever reason signed up for their care and helps them with everything, right from encouraging them to get up and take a shower to helping them with mobility issues, transfer issues, etc. Depending on the level of care, their medical functioning, or psychiatric needs, a large gap in the care of our older inmates is filled in by the peer caregivers, who are basically other offenders.” (MP34)*

Underlining why it is ethically questionable to rely on peer caregivers to provide medical care for older inmates, our participants succinctly pointed to inappropriate behaviours some peer-caregivers show towards the older inmates. For example, they underlined their scepticism towards the ways the peer-helpers treat the older inmates, referring to incidences of bullying and other unfortunate experiences the older inmates faced. Thus, without disregarding its benefits, participants described concerns in relation to the conditions under which the peer-support program could facilitate the abuse and mistreatment of mentally ill older adults in the prison settings.

*P: “There are benefits of integrating some of our older guys with higher functioning offenders who become their peer caregivers. But I also think that sometimes it is not appropriate to have some of our older, more vulnerable offenders on the unit, with younger inmates who are really not acting appropriately.” (MP20)*

*I: “Okay. For what reason do you sometimes think it is not appropriate?”*

*P: “Well we have incidences of the younger guys bullying some of the older offenders or taking advantage of them or doing many other things. Many things happen.” (MP20)*

Some of our participants, lastly, commented on the motivation of some of the offenders for signing up to become caregivers. This was also discussed in relation to why it is ethically questionable to involve offenders as caregivers. The participants reported that some offenders become caregivers to show power and control over their peers. As one participant told to the interviewer, the peer caregivers’ motivation is a more pressing issue than them being non-professionals.

*I: “Would it be an issue that an offender you rely on is not a professional?”*

*P:” Yes. But professional background is I think less of an issue because obviously, for instance, in the community we rely on family members and other people. They are not professionals to tell us how people are doing. There is some degree of screening of these offenders who sign up to be care givers, in terms of how their behaviours have been like while they are here, or how their willingness to work had been. Those kind of things are looked at and screened, but... you cannot always be sure about their motivations and reliability.” (MP34)*

*I: “What do you think what kind of other motives they could have?”*

*P: “Well I think the other motives can vary I think and some of these motives may not have much direct impact on the older inmates they care for, which is fine. And I do not have a problem with those kind of secondary gain motives. For instance, they might want to demonstrate that in some way they are now changed, become caring persons and thus deserve earlier release. That part I do not have much problem with as it does not directly impact the care of the older offenders. Where I would have concerns is when they are motivated by issues of power and control over the older inmates, which result in some degree of mistreatment and abuse that we do not openly see and notice.” (MP34)*

Further extending the discussion of the peer caregivers’ motives, it was argued that some peer helpers use their role as caregivers for economic gain.

*“But there's like an internal law which I tell you in quotation marks. The older inmates sometimes pay their peer helpers with soft drink or some other stuff to clean the cells. Some of them say well I'm too old I'm not able to clean my floor. That's because they're going to pay. It is really not us who manage all these. There are some peer caregivers who abuse the older inmates by asking them a lot in exchange for their services.” (MP22)*

## Discussion

The findings of this study carry implications for health care for mentally ill older adults in prison settings, including geriatric mental health care. The findings reflect on ethical issues of dignity and vulnerability of incarcerated older adults with mental illness in Canada. Considering the importance of researching health provider perspectives for better public health [31, 50–52], this study draws on experiences of mental health professionals to discuss ethical implications pertaining to geriatric mental health in prisons. Analysing the data from interviews with 34 mental health professionals in selected correctional facilities in Canada, we found that these participants reported to have experienced the following challenges: (1) prisons being not suitable places for older adults; (2) imprisoned older adults needing emotional care; (3) inadequate housing conditions; (4) lack of manpower and relevant expertise; and (5) ethical concerns regarding the peer-support program. Despite the heterogeneity of participants’ training and work experience, most of these experiences were mostly held across all participants. By highlighting the views and experiences of the participants in our study, we underline the ethical challenges in prison mental health care for older adults in Canada and provide a few recommendations to address the highlighted problems.

Incarcerated older adults with special care needs is one of the main themes reported in this study. It was often argued that prisons are not a place for older persons. This can be interpreted into two ways. First, our findings illustrate that keeping older adults within prison walls is by itself ethically questionable whether the prison facility is adequate or not. There has been an ongoing debate over the imprisonment of older people. “Conflicts between the medical and custodial treatment of prisoners” are documented decades ago as serious prison ethical concerns in Canada [53], and this concern is greater in the case of older offenders in particular [54]. Incarceration, by its very nature of being a punishment, interferes with dignity, quality of life and healthy ageing [6, 55]. The new and complex physical environment of a prison creates a challenging setting for older persons, and the moral implications of incarcerating older persons are thus worth pondering [54].

Second, our findings also add that prison facilities are inadequate to meet the health needs of incarcerated older individuals and this makes imprisoning older adults even more unethical. The debate over whether older adults should be imprisoned is exacerbated by the fact that correctional facilities were never designed to be nursing homes or geriatric wards for persons with age-related health conditions. As it is a legal duty in Canada [56], it is also ethically important to treat incarcerated adult with respect for their dignity that morally oblige to providing them with adequate care. As Filinson postulates in a study conducted among 67 male inmates in the US, life in custody is not suitable to promote health and successful ageing [57]. Our findings concerning the suitability of prisons for older adults accords with what Canada’s Office of the Correctional Investigator asserts: “Prison is not the appropriate environment to provide end of life care. Hospice and palliative care are specialized services and should not take place in a prison setting.” [20]. As noted by some mental health professionals in this study, the complex health conditions and needs of older persons in prisons add to the concern about the inadequacy of many prison facilities in Canada. Older persons residing in the correctional facilities included in this study were often described as lower functioning individuals needing extensive, 24-h health care. Typical health conditions mentioned in particular are mental health problems such as dementia – a finding that is consistent with literature that describes the health of incarcerated older persons as mostly characterised by dementia and other mental health conditions [2, 58].

The complexity of incarcerated older adults’ health conditions was further elaborated by referring to the co-existence of somatic health conditions and mental illnesses. When somatic health problems such as cancer, diabetes, respiratory problems, heart conditions and physical impairments merge with mental health challenges, the health conditions and care needs of incarcerated older persons become more complex. Similarly, a study with correctional health care providers in the US shows that mental illness among older incarcerated persons frequently exacerbates their physical conditions and vice versa, thereby posing a challenge to correctional health care providers in the face of resource limitations [31].

In addition to physical and mental health care, incarcerated older adults also require emotional support and positive relationships. Evidence shows that older adults need more psychological care and better benefit emotionally from supportive relationships [30, 59]. For some older persons, a warm environment that feeds the mind is even considered far more valuable than foods for the belly [60]. The mental health professionals in this study highlighted their worries in case imprisoned older adults who feel lonely and miss their old networks commit suicide or do harm upon others unless they get the needed emotional support and build their social relationships. In line with this concern, previous studies showed that suicidal ideation is mostly associated with weak social connections and depression resulting from, among other things, missing old connections and social life even after decades of incarceration [61, 62]. Even though prison misconduct is inversely related to the offender’s age [2] and suicidal attempts are less common among older inmates as compared with younger offenders [63], suicidal ideation is rather currently more common among older prisoners and it is anticipated that suicide among the ageing population will be an increasing problem in the future [63, 64].

Our findings also indicate that correctional facilities lack the resources required to meet the health care needs of older adults. One example is housing conditions that are not compatible with, for instance, the physical health conditions of older imprisoned offenders. Due to their small size, the cells were described as inconvenient for both older adults and their carers. Such resource scarcity and the need for prioritisation are known to be one of the main factors for increasing ethical dilemmas and the need for ethical competence among health professionals [65, 66]. As incarcerated older persons often report more health conditions than younger prisoners [67], the convenience of prisons’ physical environment is more important for this population and would need better funding and expertise to be put forth. In addition to the inadequacy of housing conditions, our findings illustrate grave human resource constraints, which can be seen in two ways. First, there is shortage of manpower to provide the needed mental health care services in prisons for older incarcerated persons. The disproportion between human resource and workload interferes with the provision of crucial mental health services such as, for example, cognitive assessment and counselling. Our findings illustrate that since there are no enough mental health professionals, there is no time to provide such mental health services in good quality. Older imprisoned adults need more of care providers’ time and meeting this need is challenging for care providers given their tight work schedule [31]. Second, there is lack of relevant knowledge to provide ethically competent care to incarcerated older persons with mental health issues. As indicated in our findings, it is obvious that lack of expertise is directly linked with the availability of manpower – meaning the higher the number of experts, the greater the chance for more relevant knowledge. However, the availability of manpower does not always guarantee the availability of enough and relevant expertise, especially in the case of geriatric mental health care in prison settings where highly specialised knowledge and competence are crucial [34, 68]. The lack of expertise reported in our study to handle mental health issues such as, for example, dementia, trauma and personality disorders pose a serious ethical concern and warrant attention from prison authorities in Canada. Similarly, a recent systematic review underlines the existing lack of mental health expertise among prison staff to provide adequate care to incarcerated older adults in UK, France and USA, and notes the need to deliver sufficient staff training [35]. Since incarcerated older adults did not participate in this study, it was not possible to discuss and come up with strong conclusions about the consequences of residing in an inadequate prison environment on the lives of older adults. However, from the perspectives of the mental health professionals in our study, it is arguable that the prison environment, instead of adequately meeting their health care needs, rather puts older persons in a more complicated and unfortunate life condition.

Lastly, our findings illustrate ethical challenges in involving peer offenders in the care of incarcerated older persons with mental health issues. Without disregarding the benefits and strengths of the peer-support program, it should be pointed out that this program poses ethical issues. For example, involving offenders to care for a sensitive prison population – mentally ill geriatric offenders – necessitates careful ethical considerations. As our participants noted, the competence and motives of peer caregivers can be worrying, as evidenced by instances of peer support turning into peer abuse and mistreatment. This worry found in our results is noteworthy since, as evidence demonstrates, older persons in custody report bullies and abuses inflicted by younger prisoners [59]. Ensuring a safe and secure prison environment for incarcerated older adults is usually a challenge [2] and it is evident that their relationship with younger peer offenders can contribute to this.

As the argument about whether it is permissible to incarcerate older people continues, as also indicated in the findings of this study, the immediate and most convenient solution is to improve the prison environment to address the health care needs of older persons in general and those with mental illness in particular [54]. Regardless of the crimes older people commit, it is much preferred that ensuring respect of dignity and care for incarcerated older persons remains a priority [54]. In the face of an ageing society, Correctional Service Canada should be more prepared to deal with the ‘graying of Canada’ in Canadian prisons [69]. Specialised interventions are necessary to manage and improve mental health conditions of incarcerated older adults, as well as reduce burden on criminal justice system [1]. As “mental illness in older prisoners is a result of complex interactions between numerous individual and environmental factors” [34], efforts should be made to improve prison environments. Prisons have the capacity to serve older persons as an escape from loneliness, delinquency and even poverty, as well as a place of respect and accomplishment [6]. As indicated in the findings of the current study, despite its challenges, the peer-support program serves as an important strategy to improve the prison environment for incarcerated older persons. Previous studies evidenced the benefits of prison-based peer programs for the wellbeing of incarcerated persons. For example, a study among female inmates in the US reports that peer-support programs created supportive relationships and emotional support for female inmates, thereby helping them overcome depression and cope with the pains of incarceration [70]. In Canada as well, there is evidence that peer-support programs are useful especially for the psychological, emotional and social wellbeing of mentally ill inmates [71]. There are indeed favourable sides to ageing in custody [6, 72–74], and more strategies are needed to build on existing opportunities.

## Limitations

Even though our study provides interesting and new insights in the area of geriatric mental health care in correctional facilities, it is not without limitations. One most important limitation lies in the fact that it was limited to health professionals as we only aimed at recruiting this group in Canada. The inclusion of perspectives of incarcerated older adults with mental illness might have brought more perspectives to the research question. For our overall project, we did recruit older persons with mental health illness in Switzerland [75, 76]. Doing the same in Canada was beyond our research scope. An additional limitation is perhaps that the interviews were one-off, potentially constraining possibilities for broader discussion of some ethical issues with research participants. Employing a repeat-interview design might have also increased chances of developing stronger trust with participants. However, as the interviews were conducted by trained researchers, we believe that good rapport was built to collect data rich enough to meet the study’s objective.

## Conclusions

In this study, we conducted interviews with mental health professionals in Canada to investigate ethical concerns in the care of mentally ill incarcerated older adults. This study has found that prisons are not suitable places for older adults in general and for those with mental health issues in particular, mainly because of the complex health care needs of imprisoned older adults and the lack of relevant resources in correctional facilities. As highlighted by the pandemic of COVID-19, financing health care in prison seems also secondary, thereby leading to an important ethical question of who should have the funding. Our findings pose ethical concerns that necessitate much attention from stakeholders. The findings can also inform preparedness, policy and rehabilitation efforts tailored to incarcerated older adults with mental illness. Future efforts to study geriatric mental health care should diversify recruitment to involve informal and/or voluntary caregivers of incarcerated older adults. Moreover, this study can provide the bases for further qualitative and quantitative studies of ethical challenges in involving peer offenders in the care of mentally ill incarcerated older adults in Canada and worldwide.

## Supplementary Information

**Additional file 1.** Interview guide

## Data Availability

The dataset analysed for this study is not publicly available due to the risk of compromising individual privacy of study participants. However, parts of the transcripts relevant for this paper can be obtained from the corresponding author upon reasonable request.

## Abbreviations

CSC: Correctional Service Canada
