# Supplementary material for: Challenges in providing ethically competent health care to incarcerated older adults with mental illness: a qualitative study exploring mental health professionals’ perspectives in Canada
Source: BMC Geriatr. 2021 Dec 18;21:718. doi: 10.1186/s12877-021-02687-9 (PMC8683829; doi:10.1186/s12877-021-02687-9)
Supplement: Supplementary file 1 — Additional file 1. Interview guide [file 12877_2021_2687_MOESM1_ESM.docx]

| **Interview guide / theme / technique** | **Interview questions** |
| --- | --- |
| **Introduction** | 1. What is/was your motivation to work in the field of mental health care for offenders? 2. What work experience do you have in this field? 3. What involves your current position? |
|  | 1. In which setting do you mostly work with incarcerated older offenders? |
| **Mental Health Care** | 1. Could you briefly summarize, the way mental health care is organized in your institution?    1. What are the characteristics of the treatment?    2. What is the general frequency of these treatments (per week or month), and how long do they last?    3. How is access to a MH care professionals guaranteed in case of unexpected episodes? |
|  | 1. Are the treatments provided for older offenders the same?    1. Does the treatment for older people differ in any way?    2. Do you notice any change in your attitude towards older adults?    3. If you could change anything about the currently provided interventions to make it most suitable for older detainees, what would you do? 2. Could you name the three most common needs that you have noticed treating older offenders? 3. What would you do to address these needs? |
| **Access to Mental Health Care** | 1. Could you explain the process of how a decision is made that an offender gets in touch with mental health care staff? |
| **Role Conflict** | 1. Elicitation Technique:   Where would you position yourself? |
